# Multi-Step Enzymatic Production and Purification of 2-Keto-3-Deoxy-Galactonate from Red-Macroalgae-Derived Agarose

**DOI:** 10.3390/md20050288

**Published:** 2022-04-25

**Authors:** Sora Yu, So Young Park, Dong Hyun Kim, Eun Ju Yun, Kyoung Heon Kim

**Affiliations:** 1 Department of Biotechnology, Graduate School, Korea University, Seoul 02841, Korea; sora90715@korea.ac.kr (S.Y.); thdud2502@naver.com (S.Y.P.); 2 Department of Marine Food Science and Technology, Gangneung-Wonju National University, Gangneung 25457, Gangwon, Korea; dhkim85@gwnu.ac.kr; 3 Division of Biotechnology, Jeonbuk National University, Iksan 54596, Korea; 4 Department of Food Bioscience and Technology, College of Life Sciences and Biotechnology, Korea University, Seoul 02841, Korea

**Keywords:** keto-deoxy-sugar, 2-keto-3-deoxy sugar acid, 2-keto-3-deoxy-l-galactonate, agarose, red algae, 3,6-anhydro-l-galactose

## Abstract

2-keto-3-deoxy sugar acids, which have potential as precursors in medicinal compound production, have gained attention in various fields. Among these acids, 2-keto-3-deoxy-l-galactonate (KDGal) has been biologically produced from D-galacturonate originating from plant-derived pectin. KDGal is also found in the catabolic pathway of 3,6-anhydro-l-galactose (AHG), the main component of red-algae-derived agarose. AHG is converted to 3,6-anhydrogalactonate by AHG dehydrogenase and subsequently isomerized to KDGal by 3,6-anhydrogalactonate cycloisomerase. Therefore, we used the above-described pathway to produce KDGal from agarose. Agarose was depolymerized to AHG and to agarotriose (AgaDP3) and agaropentaose (AgaDP5), both of which have significantly higher molecular weights than AHG. When only AHG was converted to KDGal, AgaDP3 and AgaDP5 remained unreacted. Finally, KDGal was effectively purified from the enzymatic products by size-exclusion chromatography based on the differences in molecular weights. These results show that KDGal can be enzymatically produced and purified from agarose for use as a precursor to high-value products.

## 1. Introduction

Keto-deoxy sugars are known to possess potential as precursors for producing medicinal compounds [1]. In particular, the demand for 2-keto-3-deoxy sugar acids, which are key intermediates of the central metabolic pathways of hexose and pentose and integral constituents of bacterial polysaccharides, have been continuously increasing in use in various fields to elucidate the mechanisms involved in microbial metabolic processes and to produce biochemicals using metabolic engineering [2]. However, there have been few attempts to produce 2-keto-3-deoxy-sugar acids such as 2-keto-3-deoxy-D-gluconate, 2-keto-3-deoxy-l-gaclatonate (KDGal), 2-keto-3-deoxy-D-xylonate, and 2-keto-3-deoxy-l-arabinonate [2,3,4]. These sugar acids can be produced using either chemical or biological methods. Biological methods are known to be advantageous over chemical methods because the latter often require many reaction steps and involve non-stereoselective reactions, which result in racemic product mixtures and lower yields [2]. On the contrary, biological processes involve fewer reaction steps, high specificity, and enantioselectivity and are therefore preferred over chemical processes for the synthesis of 2-keto-3-deoxy-sugar acids.

KDGal has been biologically synthesized from galacturonate derived from pectin present in plant biomass [4]. Pectin is a group of galacturonate-rich polysaccharides [5]. However, galacturonate residues, the main backbone of pectin, are known to be highly methyl-esterified [6]. In addition, pectin is considered to be the most complex polysaccharide, which complicates the saccharification process with additional steps, such as de-methyl-esterification in order to obtain galacturonate [7,8]. However, KDGal, an intermediate in the galacturonate catabolic pathway in fungi [9], has also been discovered in the metabolic pathway of 3,6-anhydro-l-galactose (AHG) of a marine bacterium recently [10]. AHG, a major monomer comprising agarose, which is a linear polysaccharide in marine biomass [11], is oxidized to 3,6-anhydrogalactonate (AHGA) and subsequently isomerized into KDGal [10]. Thus, AHG derived from agarose in marine biomass can be used as a substrate for producing KDGal. However, even though agarose has a simpler structure than that of plant biomass pectin, marine biomass agarose, unlike plant biomass, has not been utilized for producing KDGal yet [4]. In this study, we attempted to produce KDGal using agarose from marine biomass via a biocatalytic enzyme-mediated process.

**Figure 1:**
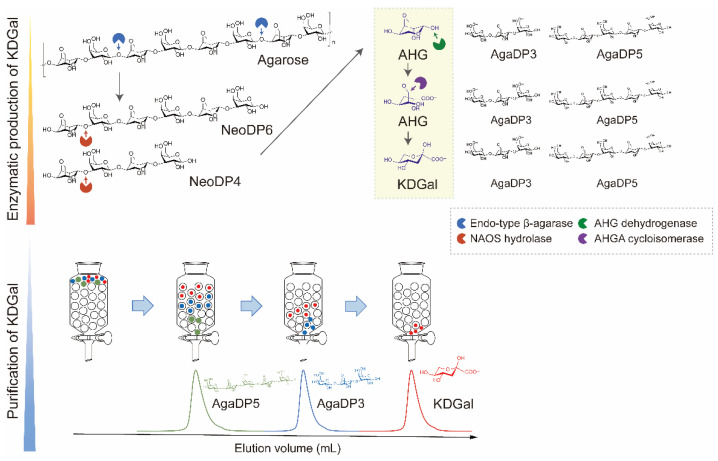
Scheme of KDGal production from agarose. Agarose can be degraded into AHG by two-step enzymatic hydrolysis using endo-type β-agarase and NAOS hydrolase. Subsequently, AHG is converted into KDGal by a two-step enzymatic reaction using AHG dehydrogenase and AHGA cycloisomerase. KDGal can be purified by size-exclusion chromatography using water as an eluent. Abbreviations: AHG, 3,6-anhydro-l-galactose; AHGA, 3,6-anhy-drogalactonate; KDGal, 2-keto-3-deoxy-l-gaclatonate; NAOS; neoagarooligosaccharide.

In this study, we designed a three-step method for the production of KDGal from agarose: (1) enzymatic production of AHG and neoagarooligosaccharides (NAOSs) from agarose, (2) enzymatic production of KDGal from AHG, and (3) purification of KDGal from the reaction product mixture (Figure 1). Agarose is the most abundant polymer in red macroalgal biomass and has gained much attention as a sustainable source owing to its higher carbohydrate and lower lignin content compared to that of plant biomass [12,13]. AHG and D-galactose form agarose via alternative α-1,3- and β-1,4-glycosidic bonds [11]. Thus, monomeric sugars such as AHG and D-galactose can be produced by the enzymatic hydrolysis of agarose using β-agarase and α-neoagarooligosaccharide hydrolase (NAOH) (Figure 1). Accordingly, agarose can be hydrolyzed into NAOSs, including neoagarotetraose (NeoDP4) and neoagarohexaose (NeoDP6), by endo-type β-agarase. NAOH can then act on NeoDP4 and NeoDP6, leading to the production of AHG and agarotriose (AgaDP3) and AHG and agaropentaose (AgaDP5), respectively [14]. AHG can be subsequently converted to KDGal using the enzymes AHG dehydrogenase (AHGD) and AHGA cycloisomerase (ACI), which, respectively oxidize AHG into AHGA and isomerize AHGA into KDGal, found in the AHG catabolic pathway of *Vibrio* sp. strain EJY3 (Figure 1) [10]. Finally, high-purity KDGal can be obtained through a purification step using size-exclusion chromatography owing to the large differences in molecular weights between KDGal and the by-products, AgaDP3 and AgaDP5 (Figure 1). In addition, agarooligosaccharides (AOSs), including AgaDP3 and AgaDP5, which remain after KDGal separation, are known to have prebiotic, antioxidant, and anti-inflammatory activities [15,16,17,18,19]. Thus, even byproducts produced along with KDGal can be utilized as high-value-added products in this process. Overall, this study showed that agarose-containing red macroalgal biomass can potentially be used in the production of high-value chemicals, including KDGal.

## 2. Results

### 2.1. Enzymatic Depolymerization of Agarose into AHG and AOSs

**Figure 2:**
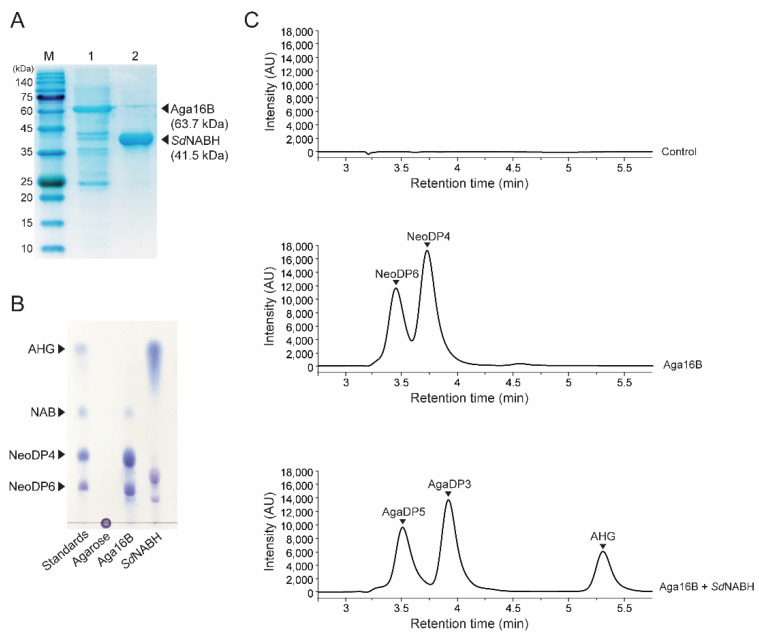
Enzymatic production of AHG from agarose. (**A**) SDS-PAGE analysis of the purified recombinant Aga16B and *Sd*NABH. Lanes: M, protein markers; 1, Aga16B; 2, *Sd*NABH purified by affinity chromatography. (**B**) TLC analysis of serial enzymatic reaction products of Aga16B and *Sd*NABH with agarose for producing AHG. (**C**) HPLC analysis of serial enzymatic reaction products with agarose for producing AHG. Abbreviations: AHG, 3,6-anhydro-l-galactose; AgaDP3, agarotriose; AgaDP5, agaropentaose; NAB, neoagarobiose; NeoDP4, neoagarotetraose; NeoDP6, neoagarohexaose; TLC, thin-layer chromatography; SDS-PAGE, sodium dodecyl sulfate-polyacrylamide gel electrophoresis.

To obtain AHG as the substrate for producing KDGal from agarose saccharification, recombinant endo-type β-agarase, Aga16B, and NAOH, *Sd*NABH were purified. The purified Aga16B and *Sd*NABH were identified by SDS-PAGE based on their theoretical molecular weights of 63.7 kDa and 41.5 kDa, respectively (Figure 2A). Agarose was initially hydrolyzed mainly into NAOSs, specifically NeoDP4 and NeoDP6, via enzymatic reaction using Aga16B, as described previously [20] (Figure 2B,C). The subsequent enzymatic reaction of *Sd*NABH using Aga16B reaction products containing NeoDP4 and NeoDP6 produced AHG, AgaDP3, and AgaDP5 as reaction products, as previously described (Figure 2B,C) [21]. These results were also confirmed by HPLC analysis. In the analysis of products formed by Aga16B, two peaks corresponding to NeoDP4 and NeoDP6 were detected, and in the analysis of *Sd*NABH reaction products, three peaks corresponding to AHG, AgaDP3, and AgaDP5 were detected, which is consistent with results obtained upon TLC analysis (Figure 2B,C).

### 2.2. Enzymatic Production of KDGal from AHG

**Figure 3:**
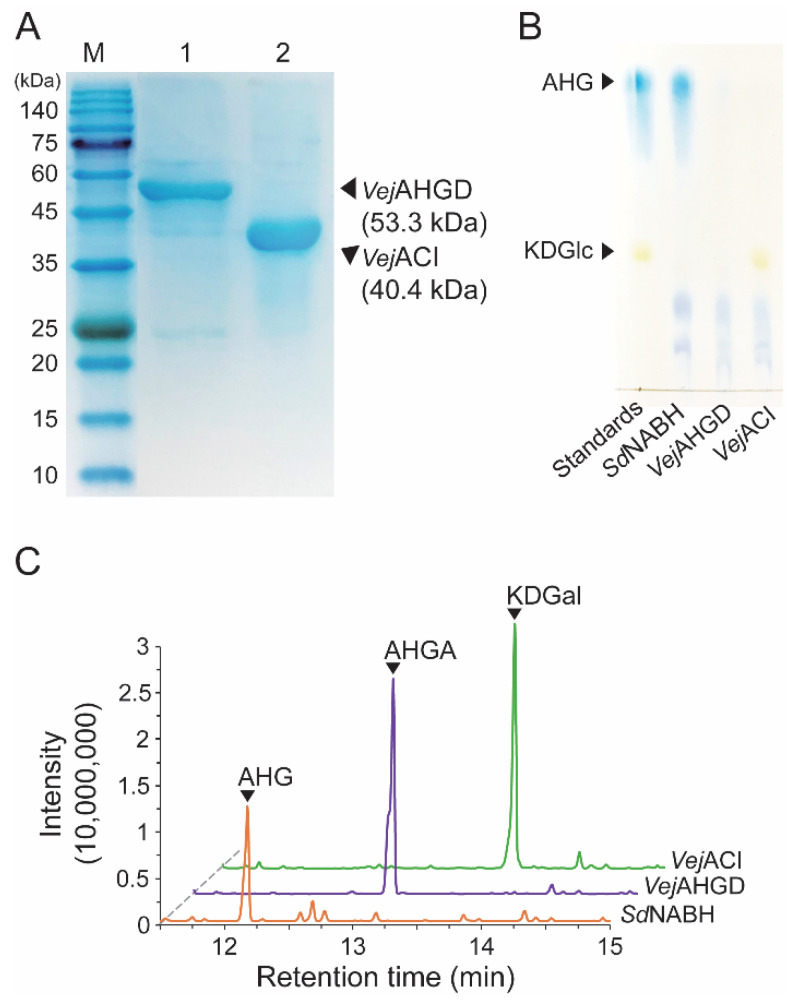
Enzymatic production of KDGal from AHG. (**A**) SDS-PAGE analysis of the purified recombinant *Vej*AHGD and *Vej*ACI. (**B**) TLC analysis of serial enzymatic reaction products of *Vej*AHGD and *Vej*ACI with *Sd*NABH reaction products for producing KDGal. (**C**) GC-MS total ion chromatograms of serial enzymatic reaction products of *Vej*AHGD and *Vej*ACI with *Sd*NABH reaction products. Abbreviations: AHG, 3,6-anhydro-l-galactose; AHGA, 3,6-anhydrogalactonate; KDGal, 2-keto-3-deoxy-l-gaclatonate; KDGlc, 2-keto-3-deoxy-D-gluconate; GC-MS, gas chromatography–mass spectrometry; TLC, thin-layer chromatography; SDS-PAGE, sodium dodecyl sulfate-polyacrylamide gel electrophoresis.

**Figure 4:**
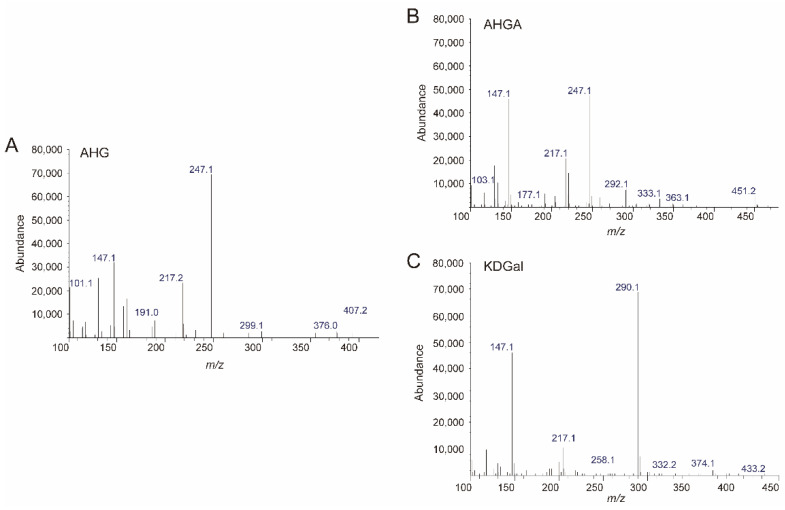
Analysis of the enzymatic reaction products of *Vej*AHGD and *Vej*ACI with AHG by GC-MS. The mass spectra of (**A**) AHG, (**B**) AHGA, and (**C**) KDGal. Abbreviations: AHG, 3,6-anhydro-l-galactose; AHGA, 3,6-anhydrogalactonate; KDGal, 2-keto-3-deoxy-l-gaclatonate; GC-MS, gas chromatography–mass spectrometry.

Because we prepared *Sd*NABH products containing AHG, two-step enzymatic reactions were performed to convert AHG to KDGal. *Vej*AHGD and *Vej*ACI, originating from *Vibrio* sp. EJY3, which has been reported to metabolize AHG as the sole carbon source, were overexpressed in *E. coli*, purified, and identified by SDS-PAGE based on their theoretical molecular weights of 53.3 kDa and 40.4 kDa, respectively (Figure 3A). We confirmed that AHG was converted to AHGA, an intermediate of the AHG metabolic pathway in *Vibrio* sp. EJY3 by *Vej*AHGD (Figure 3C). Subsequently, we also observed that a peak corresponding to KDGal appeared, whereas a peak corresponding to AHGA disappeared after incubation with *Vej*ACI using GC-MS. In addition, the production of AHGA and KDGal was verified by comparing the mass fragmentation patterns of AHG, AHGA, and KDGal that were produced in this study with those observed in a previous study [10] (Figure 4). In particular, the generation of unique daughter ions at 247 and 290 *m*/*z* for AHGA and KDGal, respectively, was observed in the GC-MS analysis (Figure 4B,C).

While the peak corresponding to AHG disappeared, the peaks corresponding to AgaDP3 and AgaDP5 were observed in TLC analysis (Figure 3B).

### 2.3. Purification of KDGal from the Enzymatic Reaction Product Mixture

**Figure 5:**
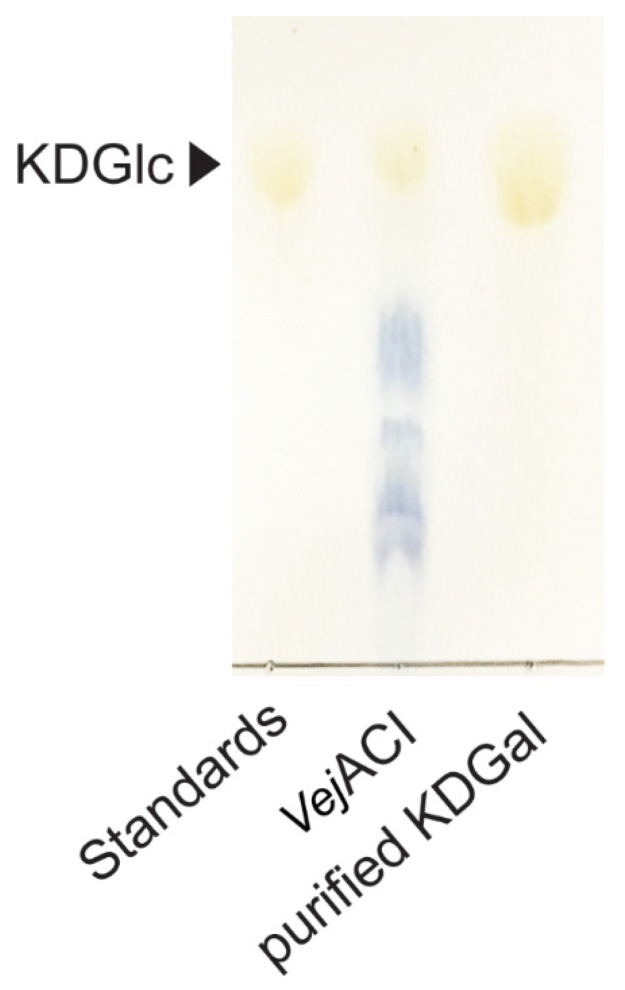
TLC analysis of purified KDGal from reaction products of *Vej*ACI. Abbreviations: KDGal, 2-keto-3-deoxy-l-gaclatonate; KDGlc, 2-keto-3-deoxy-D-gluconate; TLC, thin-layer chromatography.

The purification process is essential for obtaining high-purity KDGal. After the enzymatic reaction with *Vej*ACI, the reaction product mixture contained KDGal along with AgaDP3 and AgaDP5, which are the products of the NABH reaction with NeoDP4 and NeoDP6, respectively (Figure 3B). Molecules with different molecular weights can be separated based on their size as they pass through a size-exclusion chromatography column. Because there is a large difference between KDGal, with a molecular weight of 178.14 g/mol, and AgaDP3 and AgaDP5, with molecular weights of 486.4 and 792.7 g/mol, respectively, KDGal was able to be easily separated from the mixture containing AgaDP3 and AgaDP5 using size-exclusion chromatography (Figure 5).

## 3. Discussion

KDGal, a keto-deoxy sugar that has the potential to be used as a precursor in pharmaceutical production, is found in the catabolic pathway of AHG, which is one of the major monomeric sugars in red macroalgal agarose. As a proof of concept, we have demonstrated that KDGal, produced from plant-derived D-galacturonate so far, can also be produced from marine macroalgal agarose in this study.

Prior to KDGal production, AHG was produced with AgaDP3 and AgaDP5 from agarose by enzymatic hydrolysis. For depolymerization of agarose, the unique properties of agarose should be considered. Agarose can exist in either the sol or the gel state. Agarose in the gel state has lower accessibility to enzymes than agarose in the sol state. Thus, it is important to maintain agarose in the sol state to saccharify it efficiently, which means that agarose saccharification should be performed above the sol–gel transition temperature of agarose [20]. In addition, the near-insolubility of agarose hinders its enzymatic depolymerization. Therefore, initial pretreatment is necessary to increase the solubility of agarose in water. Chemical pretreatment of agarose has been reported to have several drawbacks, including the formation of salts and the necessary use of additional enzymes for complete depolymerization of agarose into monomeric sugars [22,23,24]. Moreover, it has been reported that AHG residues seem to be unstable under the mild acid conditions, leading to overdegradation of AHG to 5-hydroxymethylfurfural, ultimately leading to a lower yield of KDGal [25]. Thus, to overcome these drawbacks, enzymatic liquefaction, which can replace chemical pretreatment, has been suggested [20]. Therefore, the importance of thermostable ento-type β-agarases in the depolymerization of agarose has been highlighted previously [20].

Thus far, β-agarase has been the most reported and characterized agar-degrading enzyme. Aga16B is an enzyme with an optimal temperature of 60 °C, the highest temperature among those β-agarases that produce NeoDP4 or NeoDP6 as the major end products [26]. In addition, we selected Aga16B for agarose hydrolysis because Aga16B produces NeoDP4 and NeoDP6 as the main products and rarely produces neoagarobiose (NeoDP2). In the NAOH reaction, NeoDP2 is degraded into AHG and D-galactose, which have a small difference in size, making the final KDGal purification step using size-exclusion chromatography difficult. Therefore, considering the final purification process, Aga16B, an endo-type β-agarase that does not produce NeoDP2, is an appropriate choice for saccharifying agarose.

KDGal production was finally achieved using a two-step enzymatic reaction carried out by *Vej*AHGD and *Vej*ACI. Regarding KDGal production from *Sd*NABH enzymatic reaction products containing mainly AHG, AgaDP3, and AgaDP5, AgaDP3 and AgaDP5 remained after two-step enzymatic reactions of *Vej*AHGD and *Vej*ACI, while AHG was converted to KDGal (Figure 3). *Vej*AHGD has been revealed to display very high substrate specificity toward AHG [27]. While most aldehyde dehydrogenases are known to have broad substrate specificities toward various aldehyde substrates, *Vej*AHGD showed almost no activity toward other aldehyde sugars, including D-galactose, which has been associated with the unique structure of its substrate, AHG, comprising a bridged bicyclic structure [27]. Although the purification process for separating AHG from AgaDP5 and AgaDP3 in KDGal production was omitted, owing to the high substrate specificity of *Vej*AHGD, only the desired reaction, that is, oxidation of AHG without additional byproducts, was achieved. This is advantageous in reducing the number of overall processing steps by omitting the purification step prior to *Vej*AHGD reaction.

The purification process was essential since the reaction product mixture contained KDGal along with AgaDP3 and AgaDP5 after the enzymatic reaction with *Vej*ACI. We separated KDGal from the remaining AgaDP3 and AgaDP5 using size-exclusion chromatography with distilled water as the eluent. To simplify the purification step, agarose was intentionally hydrolyzed into NeoDP4 and NeoDP6 using Aga16B. If agarose had been hydrolyzed into NeoDP2, it would have been degraded into AHG and D-galactose by *Sd*NABH. The final enzymatic reaction mixture then would have contained KDGal and D-galactose, which have similar molecular weights, thereby making it much more difficult to purify KDGal from the enzymatic reaction mixture containing D-galactose using size-exclusion chromatography. Therefore, in this case, although the hydrolysis of agarose into NeoDP4 and NeoDP6 would result in a lower yield of KDGal than that obtained by decomposing agarose into NeoDP2, it has the advantage of being a simpler purification process. Another advantage is the AOSs, including AgaDP3 and AgaDP5 obtained as byproducts; they are known to have prebiotic, antioxidant, and anti-inflammatory activities and can therefore be utilized as high value-added products [15,16,17,18,19].

In addition to these advantages, the use of water, a non-toxic solvent as an eluent also provides an advantage in the purification step of this KDGal production process, whereas toxic solvents, such as methanol and chloroform, have been used as eluents for adsorption chromatography for the purification of AHG, which is the substrate for KDGal production [28]. This makes the purification of KDGal simpler and safer.

This study shows that agarose, one of the major carbohydrate components of red macroaglae, can also be used for the production of KDGal via enzymatic reactions and a simple purification process.

## 4. Materials and Methods

### 4.1. Overexpression and Purification of Recombinant Proteins

To produce KDGal from agarose, four recombinant enzymes were used: endo-type β-agarase (Aga16B originating from *Saccharophagus degradans* 2-40^T^) [20], NAOH (*Sd*NABH originating from *S. degradans* 2-40^T^) [21], AHGD (*Vej*AHGD originating from *Vibrio* sp. EJY3) [10,27], and AHGA cycloisomerase (*Vej*ACI originating from *Vibrio* sp. EJY3) [10]. To produce the recombinant enzymes, each plasmid encoding each protein was transformed into *Escherichia coli* BL21(DE3) by using the heat-shock method, and transformants were selected on agar plates containing 100 µg/mL ampicillin (Sigma-Aldrich, St. Louis, MO, USA). *E. coli* BL21(DE3) harboring genes encoding each of the proteins was grown at 37 °C in Luria-Bertani (LB; BD, San Jose, CA, USA) medium containing 100 µg/mL ampicillin until the mid-exponential phase of growth. Protein overexpression was induced by adding 0.5 mM IPTG (Sigma-Aldrich, St. Louis, MO, USA) for Aga16B, *Sd*NABH, and *Vej*ACI or 0.2% l-(+)-arabinose (Sigma-Aldrich, St. Louis, MO, USA) for *Vej*AHGD at 16 °C for 16 h. The cell pellet was collected by centrifugation at 6000 rpm for 30 min at 4 °C and resuspended in ice-cold Tris-HCl buffer (pH 7.4). Cell disruption was then performed using a sonicator (Branson Korea, Gunpo, Korea). The supernatant was collected by centrifugation at 16,000 rpm for 30 min at 4 °C to purify the soluble recombinant protein. The overexpressed recombinant protein was further purified via affinity chromatography using a HisTrap column (GE Healthcare Life Sciences, Piscataway, NJ, USA), followed by desalting and concentration using an Amicon ultrafiltration membrane (Millipore, Burlington, MA, USA). The protein concentration was determined using a BCA protein assay kit (Thermo Fisher Scientific, Waltham, MA, USA). The purified recombinant enzymes were identified using sodium dodecyl sulfate-polyacrylamide gel electrophoresis (SDS-PAGE) based on their theoretical molar mass.

### 4.2. Enzymatic Production of AHG from Agarose

To produce AHG as a substrate for KDGal production, we performed enzymatic depolymerization of agarose using the recombinant enzymes Aga16B and *Sd*NABH. To produce NAOSs from agarose, including NeoDP4 and NeoDP6, 4 mg of Aga16B was added to 100 mL of 2% (*w*/*v*) agarose (agarose, for molecular biology, Sigma-Aldrich, St. Louis, MO, USA) in 20 mM Tris-HCl (pH 7.4), and the reaction mixture was incubated at 50 °C overnight. To produce AHG from Aga16B reaction products, NeoDP4 and NeoDP6, 2 mg of *Sd*NABH was then added to the Aga16B reaction mixture, followed by incubation at 30 °C for 5 h.

### 4.3. HPLC and TLC Analyses

The enzymatic reaction products of Aga16B and *Sd*NABH were analyzed using high-performance liquid chromatography (HPLC; Agilent Technologies, Santa Clara, CA, USA) equipped with a Rezex ROA-Organic Acid H^+^ (8%) column (Phenomenex, Torrance, CA, USA) and a refractive index (RI) detector (Agilent Technologies, Santa Clara, CA, USA). The temperature of the column and RI detector was set to 50 °C. The column was eluted using 0.005 N H_2_SO_4_ as the mobile phase at a flow rate of 0.6 mL/min.

The enzymatic reaction products of Aga16B and *Sd*NABH were also identified using thin-layer chromatography (TLC). Each enzymatic reaction product (1 µL) was loaded onto silica gel 60 TLC plates (Merck, Darmstadt, Germany), which were developed with a mobile phase consisting of n-butanol, ethanol, and water (3:1:1, *v*/*v*/*v*). The plate was visualized using 10% (*v*/*v*) H_2_SO_4_ and 0.2% (*w*/*v*) 1,3-dihydroxynaphthalene in ethanol, as previously described [28].

### 4.4. Enzymatic Production of KDGal from AHG

Two recombinant enzymes, *Vej*AHGD and *Vej*ACI, were used to convert AHG to KDGal. First, to oxidize AHG into AHGA using AHGD, *Vej*AHGD was added to the NABH reaction products containing mainly AHG, AgaDP3, and AgaDP5. The AHGD reaction mixture containing 2 mM AHG, 3 mM NAD^+^ as a cofactor, and 0.05 mg/mL of *Vej*AHGD was incubated at 30 °C and 100 rpm. After 12 h, 0.05 mg/mL of *Vej*ACI was finally added to the reaction product of *Vej*AHGD to isomerize AHGA into KDGal. Subsequently, the reaction mixture was incubated at 30 °C and 100 rpm for 12 h.

### 4.5. GC-MS Analysis of KDGal

To confirm the production of KDGal, the enzymatic reaction products of *Vej*AHGD and *Vej*ACI were analyzed using gas chromatography–mass spectrometry (GC-MS). For GC-MS analysis, the reaction products of *Vej*AHGD and *Vej*ACI were derivatized as previously described [10]. Before the derivatization step, the enzymatic reaction mixtures of *Vej*AHGD and *Vej*ACI were centrifuged at 4 °C and 16,000 rpm for 5 min. Then, 20 μL of the supernatant was dried for 1 h using a speed vacuum evaporator, and methoxyamination and trimethylsilylation were subsequently performed for derivatization. Briefly, 10 μL of 40 mg/mL methoxyamine hydrochloride in pyridine (Sigma-Aldrich, St. Louis, MO, USA) was added to the dried sample and incubated at 30 °C. After 90 min, the sample was treated with 45 μL of N-methyl-N-(trimethylsilyl)trifluoroacetamide (Sigma-Aldrich, St. Louis, MO, USA) for 30 min at 37 °C. The chemically derivatized samples were analyzed using an Agilent 7890A GC/5975C MSD system (Agilent Technologies, Santa Clara, CA, USA) equipped with a DB5-MS column (30 m length, 0.25 mm diameter, and 0.25 μm film thickness, Agilent Technologies). The derivatized sample, 1 µL in volume, was injected into the GC column in the splitless mode. The oven was programmed to maintain an initial temperature of 100 °C for 3.5 min and then for the temperature to be increased to 160 °C at 15 °C/min and held for 20 min, increased to 200 °C at 20 °C/min and held for 20 min, and finally to 280 °C at 20 °C/min and held for 5 min. Electron ionization was performed at 70 eV, and the temperature of the ion source was 230 °C. The mass range was 85–700 *m*/*z*. The reaction products of *Vej*AHGD and *Vej*ACI were also analyzed using TLC, as described above.

### 4.6. Purification of KDGal from the Enzymatic Reaction Mixture

For the purification of KDGal from the enzymatic reaction mixtures, size-exclusion chromatography using Bio-Gel P-2 Gel (Bio-Rad, Hercules, CA, USA) was performed. Distilled water was used for elution at a flow rate of 0.3 mL/min. To obtain high-purity KDGal, the fractions containing only KDGal were collected and freeze-dried. The purified KDGal was finally analyzed using TLC.
